# Between Deserts and Jungles: The Emergence and Circulation of Sylvatic Plague (1920-1950)

**DOI:** 10.1080/01459740.2023.2189110

**Published:** 2023-03-24

**Authors:** Matheus Alves Duarte da Silva

**Affiliations:** Department of Social Anthropology, University of St Andrews, St Andrews, UK

**Keywords:** Amazon, emptiness, *Office International d’Hygiène Publique*, Ricardo Jorge, Southern Africa, wild rodents

## Abstract

I trace the development of the concept of sylvatic plague – the first sylvatic disease – examining its invention by Ricardo Jorge to describe a global phenomenon of plague reservoirs among wild rodents, and its circulation. The concept implied a space where plague was enzootic, and relied on a division between inhabited and uninhabited spaces and between domestic rats and wild rodents. Some of the characteristics of this space varied, but it always referred to places imagined as empty of humans and rats. In 1927, it designated ambiguously deserts, in 1935, uninhabited regions in general, and in Brazil, it referred to the jungle.

## RESUMO

O artigo retraça o desenvolvimento do conceito de peste selvática, a primeira doença selvática, da utilização por Ricardo Jorge para descrever um fenômeno global de reservatórios de peste entre roedores selvagens, até sua circulação nos anos 1930 e 40. O conceito inventou um espaço onde a peste se mantinha enzoótica, dividindo entre lugares habitados e inabitados, e roedores domésticos e selvagens. Algumas características desse espaço mudaram com o tempo, mas sempre mantendo a ideia de lugares imaginados como vazios de humanos e ratos. Em 1927, o conceito designava ambiguamente desertos, em 1935, regiōes desabitadas em geral, e no Brasil, ele se referia à selva.

The fact that some diseases affecting humans can take a sylvatic form or cycle, in other words, that their cause – be it a virus, parasite or bacterium – can be perpetuated among animals living in non-urban spaces is central to the epidemiology of a range of diseases that are transmitted from animals to humans, known as zoonoses (Jansen et al. 1999; Silva et al. 2020). The idea of sylvatic diseases was developed in the first half of the twentieth century. It was first mobilized in the late 1920s by the Portuguese doctor Ricardo Jorge (Office International d’Hygiène Publique 1926:115–117, 1927:90–91; Jorge 1927a:1271) to talk about plague (the disease caused by the bacterium *Yersinia pestis*) among wild rodents, and is still used to describe and manage this disease in places such as Brazil and the USA (Abbott et al. 2012; Brasil 2008:27). The idea of sylvatic diseases had important applications in the broader field of study of infectious diseases, with the most notable example being the case of jungle or sylvatic yellow fever in the 1930s. The discovery of a sylvatic yellow fever challenged assumptions that it was possible to eradicate it in the Americas by only focusing on the eradication of the mosquito *Aedes aegypti* in cities. In fact, the case of sylvatic yellow fever demonstrated that the virus circulated among several animals living in tropical rainforests and that it could be transmitted to humans by mosquitos other than *Aedes aegypti* (Emilio et al. 2008; Magalhães 2016:96–100).

Despite its importance in past and present, historians have not examined the development of the notion of sylvatic plague by Jorge. Nor have those who have focused on the emergence of sylvatic yellow fever discussed in depth what a sylvatic disease means in terms of a medical reasoning that takes for granted that an animal disease reservoir objectively exists in the “jungle.” To fill these lacunas, I aim to historicize the emergence and circulation of the concept of sylvatic plague in the second quarter of the twentieth century and discuss what the categorization of a disease as “sylvatic” implied for medicine, in general, and for the study of animal reservoirs in particular. I aim to show that the idea of sylvatic disease unsettled one of the most important changes brought by the so-called Pasteurian revolution: that the ontology of a disease is determined by its causative pathogen (Cunningham 1992; Latour 1984). In the case of sylvatic plague and other sylvatic diseases, what defines their identity has not been their causative pathogen (which is the same as that of their “urban” forms), but the “wildness” of the disease reservoir and the environment the animal reservoir inhabits. This study of “sylvatic plague” thus challenges the thesis that, following the bacteriological revolution, the ontology of diseases was solely determined by etiology, and argues that diseases such as plague not only continued to be understood through real or imaginary traits (clinical, demographic, racial) long-established before their bacteriological identification, but also acquired new frameworks relatively autonomous from bacteriology. In the case of sylvatic plague, these were spatial and ecological frameworks.

## Coining a global concept

Ricardo Jorge (1858–1939) was a central actor in the history of medicine in Portugal during the first half of the twentieth century. Born in Porto in 1858, Jorge graduated in medicine in 1879. In 1892, he became director of Porto’s Public Health Service, a post he occupied when he identified the presence of plague in the city in July 1899. This was the first outbreak of the disease in Portugal since the seventeenth century. In the close aftermath of the outbreak, Jorge was named General Inspector of the Sanitary Services of the Kingdom of Portugal, a position he retained after the Proclamation of the Portuguese Republic in 1910. In 1929, he became the President of the Portuguese High Council of Hygiene and, until his death in 1939, he remained a key scientific and political actor in Portugal. Jorge researched and wrote about several infectious diseases, from smallpox to Spanish flu and plague. Internationally, he represented his country at the International Sanitary Conferences of Paris in 1911–1912 and 1926, at the *Office International d’Hygiène Publique* (OIHP) from 1912 until his death in 1939, and in the League of Nations Health Organization (LNHO), from its foundation in 1924 until his death (Almeida 2013; Alves 2008; Benchimol 2013; Costa 2018). It was in two presentations within the OIHP, in October 1926 and April 1927, that Jorge proposed for the first time his scheme about what we could call the plague phenomenon. According to him, there were two “types” of plague, both caused by the same bacillus but connected to different kinds of animals: “pandemic plague” was linked to “domestic rats” and “sylvatic plague” was associated with wild rodents (Office International d’Hygiène Publique 1926:115–117, 1927:90–91).

Jorge’s propositions came at a moment when plague was a known and studied disease. It had been rediscovered as a global menace in 1894, when following the networks of the European Empires, it spread from Hong Kong to all inhabited continents, causing more than 12 million deaths, the majority of which were in India. This was the Third Plague Pandemic (1894–1959) (Arnold 1993; Chakrabarti 2012; Echenberg 2007). Since the first years of this pandemic, black and brown rats occupied an important place in a new epidemiological framing, as they were understood to be carriers and later to be reservoirs of the plague bacillus, which was transmitted to humans by rat fleas. Accordingly, anti-rat measures designed to destroy them or limit their contact with humans unfolded around the world (Silva 2016; Engelmann and Lynteris 2019; Skotnes-Brown 2023; Vann 2003).

However, in a few places, rats were not the sole villains, and wild rodents were likewise blamed for plague outbreaks. The connection between wild rodents and plague was observed in Manchuria (Lynteris 2019), South-West Russia (Jones and Amramina 2018; Jones et al. 2019), California (Honigsbaum 2016), and South Africa (Skotnes-Brown 2021). Subsequent studies led to new concepts that focused either on the species of wild rodents affected by the infection, such as “squirrel plague” in California (McCoy 1911), or on the environment occupied by the rodents, such as “steppe plague,” in the USSR which described the infection among spermophile ground squirrels, known as sousliks (Nikanoroff 1928:128), or “veld plague,” used in South Africa to frame the infection among gerbils in the veld (Pirie 1927). Seen as a whole, these studies challenged the idea of the rat as the sole protagonist in the spread of plague, and pointed out that, in those locales, the control or eradication of the disease would necessitate measures against wild rodents. These studies established that plague posed two and not one type of epidemiological problem: the common plague, or the pandemic plague in Jorge’s terms, spread by rats, and these local phenomena of plague endemicity linked to wild rodents.

Jorge’s idea of sylvatic plague drew upon these studies, but, in contrast to their local or regional focus, he claimed sylvatic plague was universal (Jorge 1928:54; Office International d’Hygiène Publique 1926:115). In Latourian terms, one could say that Jorge acted as a center of calculation, a nodal point inside a global network capable of accumulating immutable mobiles, i. e., bits of information that can travel long distances, and be combined to create new and more abstract objects, such as maps, museum collections, or equations (Latour 2003). Drawing on the networks of the OIHP, Jorge gathered scattered “bits of information” on wild rodents and plague and devised a single, standardized term, which traveled back to the places where plague among wild rodents was a problem, such as California and South Africa, and replaced the local terms (Davis 1948; Meyer and Eddie 1935). However, as discussed by Raj (2013) in his critique of Latour, such “mobiles” are almost never immutable, and they are constantly adapted, challenged, and transformed in their global circulation. As I will argue, the concept of sylvatic plague was able to act as a standard because it implied a general space where plague was perpetuated among wild rodents without the participation of humans and rats. Nevertheless, this space changed over time. In the late 1920s, this space was the “desert;” that is an environment with scarce rainfall and/or a dearth of humans and rats. In 1935, the space of sylvatic plague came to mean landscapes uninhabited by humans and by rats but inhabited by wild rodents. When the concept circulated in the late 1930s and 1940s, the notion of uninhabited places remained at the heart of new adaptations, but the idea of sylvatic plague gained local meanings. In Brazil, for instance, it evolved to mean jungle plague, a change with important practical consequences for plague management in that country (Simon 1951:25). Before elaborating on the history of the emergence and evolution of the concept of sylvatic plague, I will take Jorge’s reasoning in terms of “sylvatic” ethnographically seriously and discuss the dichotomies that he aimed to put in place with this concept.

## Where is the sylvatic?

Most if not all of Jorge’s works on sylvatic plague were written and published in French (Office International d’Hygiène Publique 1926:115–117, 1927:90–91; Jorge 1927a, 1928, 1935b). In that language, the concept appeared as *peste selvatique*. *Selvatique* was an adaptation of the Portuguese *selvático*, as explained by Jorge (1936:2): “in the Portuguese language we have *selva* […] having the same meaning of wood, forest, etc., and from *selva* comes *selvático*.” Jorge (1936:2) also emphasized that *selva* meant by extension “the Amazon, old Portuguese territory where Portuguese is the common language.” Therefore, in its first meaning, *selvático* refers to something or someone that inhabits the forests or the jungle. Nevertheless, the association with forested landscapes became an important source of criticisms of the usage of the term sylvatic plague (Girard 1948; Macchiavello 1941:68). According to these critics, the concept implied a jungle plague, but there was no such thing. In fact, the environments inhabited by the plague-infected wild rodents were mainly steppes or plains (Girard 1948:15).

However, the idea of *selvático* had a second and connected meaning, relating to a wild animal living in an uninhabited space. This second meaning was developed by Jorge in a polemic with the editors of the *American Journal of Public Health*, who suggested replacing the term sylvatic plague by wild rodent plague (Editorial 1936). Jorge (1936:4) retorted that the concept of wild rodent plague was problematic for at least two reasons. Firstly, not all wild rodents could “transmit” plague to humans, and secondly, in some places a few species of wild rodents could become “domestic,” as in the case of the multimammate mouse in South Africa (Jorge 1936:4). Therefore, concluded Jorge (1936:4), “the term ‘wild rodent plague’ has a much more extensive sense than ‘selvatic’ or ‘sylvatic plague,’ which is restricted by its definition to the foci maintained autonomous in uninhabited regions by the enzootic of certain absolutely wild species having no direct or indirect communication with human dwellings.” In sum, in Jorge’s reasoning, sylvatic plague appeared not as jungle plague, nor even as the plague of wild rodents, but instead as an enzootic among wild rodents living in wild, uninhabited places.

The idea of sylvatic plague thus put in place a double dichotomy. Firstly, a spatial one, between inhabited and uninhabited regions; a mode of thinking deeply rooted in the European imagination (Campbell et al. 2019:2). Framing a given place as empty was particularly central to European colonial expansion through the centuries, with legal terms such as *res nullius*, *territorium nullius* and later *terra nullius* being applied to populated regions of America, Asia, Africa, and Oceania to frame their human inhabitants as wild and lacking civilization and thus justify land dispossession and colonial settlement (Fitzmaurice 2007; Giminiani et al. 2021:84). This rhetoric was particularly applied to the Amazon; the so-called *selva*. Despite being populated by different indigenous people since before the Portuguese conquest, it was conceived during Portuguese colonization as the archetype of a wild and empty place (Pádua 2000:794–795). This reasoning continued in independent Brazil, with the Amazonia being described as a “land without history” or as a vast virgin landscape absent of civilization at the beginning of the twentieth century (Cunha 1966 [1909]). Reflecting this tradition, Jorge applied the imaginary of uninhabited and wild spaces, whose epitome was the Amazonian *selva*, to his concept of sylvatic plague.

Besides this dichotomy between inhabited and uninhabited places, Jorge fostered a second and connected dichotomy, this time between “domestic rats,” blamed for spreading the pandemic plague, and “wild rodents,” blamed for the sylvatic plague (Office International d’Hygiène Publique 1927:89). For domestic rats, highlighted Jorge, “one should not understand domesticated rodents, such as the rabbit and the guinea pig, but those that live in contact with the man [sic].” These were mainly the brown rat and the black rat (Office International d’Hygiène Publique 1927:89), but also included the multimammate mouse in South Africa (Jorge 1936:4). Jorge did not give more details about the differences between domesticated and domestic rats. Therefore, we can only assume that by the former he meant animals bred by humans, while the latter lived close to humans without any human intervention. The wild rodents, on the other hand, “lived in the fields [*campagne*], […] beyond the normal conditions of human existence” (Office International d’Hygiène Publique 1927:89). In arguing thus, Jorge espoused the pervasive idea, examined by Cassidy (2007:1), that the “wild is somehow out there occupying space that is untouched by human influence,” which “distorts understanding of places that are not out of time, or out of space.” In more medical terms, Jorge’s reasoning partly anticipated what is a common understanding in contemporaneous zoonosis studies: that of seeing the enzootic cycle of a given disease “as remote from humanity and existing in what is imagined as the natural and original abode of the disease” (Lynteris 2017:472). However, as I will show below, this natural space was not seen by Jorge as the place from where plague came, but the place to where plague had moved from the cities. If the idea of wild rodents suggested therefore a separation between some animals and humans, the category of domestic rats suggested an intimacy between humans, certain animals, and pathogens (Kelly and Sáez 2018). Moreover, as we will see, by arguing that some wild rodents, such as the multimammate mouse in South Africa, could become domestic and therefore be responsible for the common or “pandemic plague,” rather than to the sylvatic plague, Jorge seemed to suggest that contact with humans could ontologically transform the rodents and the type of plague they were connected to. In the following sections, I will chart how this double dichotomy appeared in Jorge’s and other scholars’ writings when they developed or adapted the idea of sylvatic plague.

## The plague of the desert

The crafting of the sylvatic plague concept is intrinsically associated with the OIHP: the first health agency with a global remit (Paillette 2012; Chiffoleau 2012:260; Cueto et al. 2019:32–36). Based in Paris and existing between 1907 and 1946, the OIHP’s main role was to keep its members updated about outbreaks of epidemic diseases – mainly cholera, yellow fever, and plague – and their prophylaxis. It functioned as an archive, a diffusor of epidemiological data, and as a consultative agency on matters of international health, being active in the formulation of the International Sanitary Conventions of 1912 and 1926 (Cueto et al. 2019:17; Paillette 2012; Sealey 2011). Concerning plague, what interested the majority of OIHP delegates in its first years was the problem of preventing its maritime circulation. Accordingly, the debates focused on measures against rats, particularly the imposition of a mandatory deratization of all ships, which was agreed at the International Sanitary Conference of Paris, in 1926 (Office International d’Hygiène Publique 1910:38–39, 1919:51, 1920:26 and 133; Ministère des Affaires Étrangères 1927:171). These debates reflected the liberal utopian vision of a world free of quarantine and of plague transmission thanks to the deployment of maritime deratization (Engelmann and Lynteris 2019).

Nonetheless, other subjects related to plague were debated within the OIHP in the 1920s, partly influenced by the arrival of new members, mainly from Africa and Asia.^1^ These new actors brought original or seldom discussed questions to the assembly, as seen in the April 1924 session. On that occasion, the South African delegate P. G. Stock presented a note written by James Alexander Mitchell, Secretary for the Public Health, and Chief Health Officer of South Africa (Office International d’Hygiène Publique 1924:20). Mitchell examined the history of plague outbreaks in South Africa in the prior 20 years, arguing that the last human cases observed in the Orange Free State (1920 and 1921) were not linked to domestic rats, but rather to wild rodents, mainly gerbils (Office International d’Hygiène Publique 1924:20–24). Mitchell’s conclusion was capitalized upon by Jorge to express his personal impression that some of “the great problems of plague were not completely solved,” such as the role played by different species of rodents and insects in the maintenance and spread of plague (Office International d’Hygiène Publique 1924:55–56). Jorge believed that the OIHP could help answer this question through a global survey relying on epidemiological data transmitted by the agency’s delegates (Office International d’Hygiène Publique 1924:56). The OIHP general assembly decided to sponsor the survey, which seemed achievable given that the agency already had experience in gathering and synthetizing similar kind of epidemiological data. Delegates were asked to forward reports on rats, wild rodents, and ectoparasites found in their respective countries and accounts of their relationship with plague (Office International d’Hygiène Publique 1924:205). From 1924 to 1926, the agency received reports covering more than 30 countries – independent states, protectorates, and colonies – which formed the basis of Jorge’s further publications (Jorge 1928).

A first draft synthesizing this material was provided by Jorge in October 1926 (Office International d’Hygiène Publique 1926:115–117). On this occasion, Jorge announced the “sketch of a new chapter in the epidemiogenesis of plague, the plague of desertic regions [*la peste des régions désertiques*], maintained by wild rodents” (Office International d’Hygiène Publique 1926:115). According to him, this plague of desertic regions existed in a few places in the world and was created by a similar process. Infected rats, wrote Jorge, “penetrating the hinterland of certain zones of Asia, Africa, and America, succeed, by the contamination of wild rodents, [which are] very sensible to the virus [sic], to ignite, in a way that is durable and independent from its origins, enzootic permanent plague hotspots [*foyers*] that we could call sylvatic [*selvatique*] because of the habitat of the wild rodents in desertic regions (steppes, veld, etc.)” (Office International d’Hygiène Publique 1926:116). These wild rodents, framed by Jorge alongside their parasites as “virus reservoirs” of plague, varied globally (Office International d’Hygiène Publique 1926:116). In South Africa, they were gerbils; in the South-West of Russia, sousliks; in Inner Asia, Siberian marmots, also known as tarbagan; and in California, ground squirrels (Office International d’Hygiène Publique 1926:116). According to Jorge, humans became infected from the sylvatic plague either by hunting these rodents, contact with them in the fields, or via small rodents that connected wild rodents with humans. In all these cases, wild rodent fleas played the role of vector. Conversely, domestic rats did not play any role in maintaining the infection among the wild rodents. To Jorge, rats “ignited” the sylvatic plague reservoir, i.e., their fleas transmitted the plague bacillus to the wild rodents, but the plague bacillus continued to circulate among the wild rodents independently from the rats. Concluding, Jorge considered that the eradication of sylvatic plague was hard to achieve. Therefore, he supported prophylactic measures that could either diminish the number of wild rodents (e.g., the protection and propagation of some “natural enemies” of rodents and the attempted creation of rodent-free zones) or prevent contact between humans and rodents, for instance, through a greater control over the tarbagan fur trade (Office International d’Hygiène Publique 1926:117).

Jorge’s first presentation received an important criticism at the assembly of the OIHP. Lucien Raynaud, the delegate of the French colony of Algeria, argued that the transmission schema devised by Jorge, where the sylvatic plague reservoir was created by infected rats, could not be completely correct, whereas a reverse contamination seemed more plausible. This hypothesis could explain, argued Raynaud, the beginning of the Third Plague Pandemic by means of the tarbagan infecting rats in Manchuria, and those, in turn, infecting other rats in Hong Kong, from where the pandemic started (Office International d’Hygiène Publique 1926:117–118). Jorge did not discard this possibility but replied that the cycle of infection started almost invariably with the rats and that wild rodents were often infected secondarily. To prove his point, Jorge used the recent case of South Africa as the ultimate example, given that the plague infection among gerbils came from rats (Office International d’Hygiène Publique 1926:119).^2^ It is worth noticing that Jorge was proposing a diametrically opposite scheme to the mainstream understanding of emerging infectious diseases (EID) in our present day (Garrett 1995; King 2004; Wald 2008). While in most of the cases, EIDs pathogens such as Ebola or SARS-CoV-2 spill over from wild animal reservoirs to humans and/or domestic animals, in Jorge’s reasoning it was the movement of rats along human infrastructures that created sylvatic reservoirs. In other words, “nature” was not seen as the origin of plague.

In April 1927, Jorge offered a more polished version of his synthesis, which was largely praised by his fellow delegates (Office International d’Hygiène Publique 1927:90–92), paving the way for the publication of the report in the Bulletin of the OIHP (Jorge 1927a, 1927b). In the published version, Jorge developed the argument about the existence of a global and integrated system of plague transmission and maintenance, represented respectively by the two “types” of plague: “pandemic” and “sylvatic” [*pandemique* and *selvatique*]. The pandemic plague was conveyed by fleas parasitizing the two species of “domestic rats.” Repeating what was common sense at the time, Jorge claimed these two rat species were great migrators and were found in almost every port and city of the world, which gave a pandemic aspect to it (Jorge 1927b:1099–1109). Sylvatic plague, on the other hand, was defined as follows: “There is a certain type of plague, from which the zoological roots and geophysical seat are different [from the pandemic plague]. Instead of domestic rodents, they are the work [*l’oeuvre*] of wild rodents. They exist not in inhabited places, but in uninhabited places. We can call epizootics among wild animals in desertic habitats [*à habitat désertique*] sylvatic plague” (Jorge 1927a:1271).

In Jorge’s schema, the epistemological unity, on the one hand of pandemic plague and, on the other hand, of sylvatic plague relied on divergent aspects. Domestic rats and their widespread presence in urban settings gave coherence to the former. The unifying factor of the latter was two-fold. Firstly, sylvatic plague worked like a reversed image of pandemic plague since domestic rats played no role in it. Secondly, sylvatic plague was unified by the locale where it existed: desert environments. In these 1926–27 usages of the concept of sylvatic plague, Jorge mixed two meanings related to the broad idea of the desert: an environment with scarce rainfall and/or an empty place. On the one hand, Jorge (1927a:1282) talked about “desert rodents” and affirmed that deserts were one of the places where sylvatic plague emerged, along with steppes and the veld (Jorge 1927a:1272). Nonetheless, he did not provide any explanation regarding why sylvatic plague seemed to emerge in desert landscapes and not in forests, for instance. On the other hand, Jorge insisted on what we could call a metaphorical idea of the desert, in the sense of an uninhabited place. Indeed, this meaning appeared in 1927 already better described, since he insisted that what characterized sylvatic plague was it being maintained among wild rodents permanently and independently from humans or domestic rats (Jorge 1927a:1272). In sum, we have here, clearly stated, the idea of a deep and remote nature where animal reservoirs of sylvatic plague existed independently from humanity.

**Figure 1.**
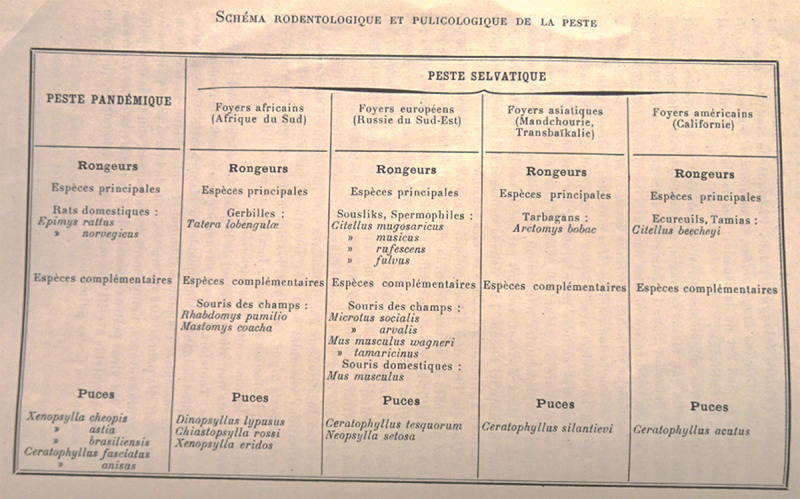
Schema rodent-logic and flea-logic of plague.

In 1928, the OIHP published Jorge’s synthesis as a book, containing pictures of the rodents and a table, named “schema rodent-logic and flea-logic of plague” (see Figure 1).^3^ The table summarized Jorge’s reasoning in terms of a division between pandemic plague and sylvatic plague. Moreover, it insisted on the existence of sylvatic plague in four hotspots (*foyers* in French) – South Africa; California; South-West Russia; and Manchuria and Transbaikalia – each of them maintained by a principal species of wild rodents and its fleas (Jorge 1928:54). The table seemed to demonstrate, firstly, that the totality of the plague phenomenon was given by its pandemic and sylvatic forms, and, secondly, that each of the forms had a different “function:” the pandemic was responsible for spreading the plague bacillus, while the sylvatic was responsible for perpetuating it in contained spaces, hence the reasoning in terms of “foyers” (Jorge 1928:54). Thus, the dream of a world free of plague thanks to an obligatory maritime deratization, which animated the debates within the OIHP and came to fruition in the International Sanitary Conference of Paris, in 1926, seemed seriously compromised by Jorge’s conclusions. Even if rats were eradicated, the table suggested, the plague bacillus would continue to exist and circulate among independent reservoirs in the wild.

## The plague of uninhabited regions

Starting in January 1932, a series of outbreaks on the border between Angola, then a Portuguese colony, and South West Africa (present-day Namibia), a former German colony under South African mandate since the end of the World War I, would challenge some of Jorge’s original categorizations of the sylvatic plague and force him to adapt his ideas. On that occasion, a plague epidemic was declared in Ovamboland, in the north of South West Africa. Amidst political and sanitary tensions created by this outbreak, Louis Fourie, an Assistant Health Officer of the South African Department of Public Health, and a former South West African Medical Officer, was sent to the Ovamboland (Thornton 1933:79–81). After some fieldwork, he affirmed that the Ovamboland epidemic was linked to a plague epizootic among migratory gerbils coming from South Africa (Fourie 1932:79). In Angola, this episode caused concern. While plague outbreaks linked to domestic rats had been occurring in its ports since 1921, a plague invasion of the southern border connected with migratory wild rodents seemed completely unexpected (Jorge 1935a:1–2). Furthermore, it affected a politically tense region – the Baixo Cunene – recently incorporated into the Portuguese Empire. Therefore, if when Jorge originally crafted the concept of sylvatic plague, he tried to name an entity whose effects seemed distant from the country and empire he represented in the OIHP, in 1932 sylvatic plague became a Portuguese imperial concern (Silva 1936).

The outbreak soon attracted international attention, and in November 1932 it was discussed during the Cape Town Sanitary Conference.^4^ At the end of this meeting, the delegates expressed the desire that African countries and colonies should inform the OIHP about plague epizootics among wild and domestic rodents occurring in their territories (La Conference du Cap 1933). In May 1933, the OIHP took notice of this. Moreover, deeming “the current situation of plague in Africa” to be dangerous, “particularly from the point of view of the infection among the rodents, wild or domestics, and of the danger of the extension of the infected zones,” the OIHP decided to proceed with another survey on plague and rodents focused only on Africa (Office International d’Hygiène Publique 1934a:830). Between 1934 and 1935, 14 colonies, protectorates, and semi-independent countries responded to this call (Office International d’Hygiène Publique 1934a, 1934b). Jorge, by this time an internationally recognized plague expert, was again chosen to synthesize the material. The first draft of the synthesis was presented in May 1935, while the official report was published in September (Jorge 1935b:1).

In the published version, Jorge repeated some previous statements and argued for the existence of two “types” of plague in Africa, one connected to domestic rats and the other with wild rodents, the sylvatic plague (Jorge 1935b:46). Concerning the latter, he first quoted his own definition (Jorge 1927a:1271, see above) but provided later in the text a significant update: “there can only be qualified as sylvatic plague, the plague that, without any relationship of dependence on the rat and the human, is conserved without division by the *vis epizootica* of wild rodents living completely apart from inhabited places” (Jorge 1935b:49). Partly informed by the migration of gerbils, this new definition did not point to any specific contained environment, such as the veld or deserts as the locus of sylvatic plague, but rather to general uninhabited places. Therefore, a sylvatic plague reservoir – at least in the case of Southern Africa – was no more confined to a given landscape, but rather appeared as a phenomenon without borders, existing, and spreading wherever humans and domestic rats were absent. Nonetheless, the Ovamboland outbreak had evidenced once again that humans could catch the plague from wild rodents, which, in some measure, challenged Jorge’s assumption that these animals lived completely apart from inhabited spaces. However, drawing on literature produced in South Africa and Angola, Jorge dodged this question by affirming that it was the multimammate mouse that connected the independent plague reservoir among wild rodents with humans, as in several localities this animal replaced the rats, living as it did in the vicinity and sometimes even inside human houses, thus becoming, to a certain measure, domestic. Therefore, plague infection in the case of the multimammate mouse was of a domestic type while the category of sylvatic plague could only apply to the infection among gerbils (Jorge 1935b:46–47). In short, in Jorge’s 1935 report, sylvatic plague appeared more than ever as the plague of non-domestic rodents.

The Ovamboland outbreak also unsettled Jorge’s original reasoning in terms of the international diffusion of the plague. One of his initial distinctions between pandemic plague and sylvatic plague was their respective international circulation. In the 1927 report, Jorge (1927a:1282) argued that the former was constantly moving around the world, while sylvatic plague was circumscribed in the four “foyers” because wild rodent migrations were “rare and occur in a reduced scale.” However, in the 1935 report, he conceded that the Ovamboland outbreak had been caused by “a sylvatic plague in motion,” whose outcome was to place the south of Angola inside “the broad South African sylvatic plague zone” (Jorge 1935b:30). The recognition that sylvatic plague reservoirs could circulate beyond international borders was echoed in the OIHP assembly in 1935. Following Jorge’s presentation, the assembly concluded that “the [African] ports are becoming more sanitized. The only worrying point is the persistence, and the progression toward the north, of the infection among the gerbils and other wild rodents in South Africa” (Comité Permanent de l’Office International d’Hygiène Publique 1935:1036). Understanding sylvatic plague as an in-motion phenomenon partly dovetailed with the more classical problem posed by the global circulation of plague via domestic rats as it acknowledged that wild rodents, by their movements, could expand sylvatic plague reservoirs. Thus, if the absence of international circulation could no longer be ascribed to sylvatic plague, what seemed to characterize the sylvatic plague was the assumption that it was perpetuated among wild rodents in uninhabited places, a position maintained by Jorge in 1935 which resonated in further adaptations of the concept.

## The plague of the jungle

From the 1930s onwards, the concept of sylvatic plague began to circulate beyond Jorge’s writings.^5^ Drawing upon Jorge’s reasoning, a Sylvatic Plague Committee, led by Karl Meyer, was created in 1935 in the USA to investigate the suspected presence of plague among rodents in several states of the West Coast (Shepard 1935:386–387; Meyer 1936:96:). The committee found a vast plague reservoir existing in uninhabited spaces and independent from rats. Given that this reservoir implied several species of wild rodents and resembled phenomena observed in South Africa and Manchuria, it was felt that replacing the former idea of squirrel plague by that of sylvatic plague was justified (Meyer and Eddie 1935:400–401).

The idea of sylvatic plague also informed important sanitary actions in the late 1930s and 1940s in places where, contrary to the USA, a plague among wild rodents did not exist. This was the case of Brazil, a country, nonetheless, historically associated with terms such as *selva* and *selvático*. The plague arrived in Brazil in 1899, principally in the capital, Rio de Janeiro (Nascimento and Silva 2013). The Brazilian Federal Government launched an anti-rat campaign, which was believed to have reduced plague cases by the early 1910s (Silva 2020:284–290). However, from the 1920s onwards, plague outbreaks occurred in medium-sized cities and rural villages, especially in the backlands, a semi-arid region of the North-East (Parreiras 1935; Silva Junior 1942). In 1939, the Pan-American Health Organization, in accordance with Brazilian authorities, commissioned the Chilean doctor Atilio Macchiavello to study this situation (Macchiavello 1941:9–10). Macchiavello (1941:69–95, 103) concluded that plague became endemic in a vast area of the backlands, where it was linked to domestic rats. He also concluded that although sylvatic plague still did not exist in Brazil, the risks of its emergence should not be ignored. Despite having read Jorge, Macchiavello (1941:107) applied a slight change in his own definition of sylvatic plague, framing it as also a problem of forested regions. Indeed, to Macchiavello (1941:40), sylvatic plague “can occur in uninhabited zones, generally jungles, bushes or mountains, where the man [sic] does not live.” Based on Macchiavello’s conclusions, the Brazilian Government created in 1941 the *Serviço Nacional de Peste* [Plague National Service] (SNP), which lasted until 1956 (Luna 2021). The SNP’s main goals were to suppress the current outbreaks linked to domestic rats, and in so doing, not only control the disease but also prevent the emergence of sylvatic plague (Castro 1942:2; 1947:316). To achieve these aims, the SNP deployed actions to stop contact between humans and rats, and between rats and wild rodents, and to destroy both rats and wild rodents (Serviço Nacional de Peste 1944; Castro 1947:316; Freitas 1988:75–76).

**Figure 2.**
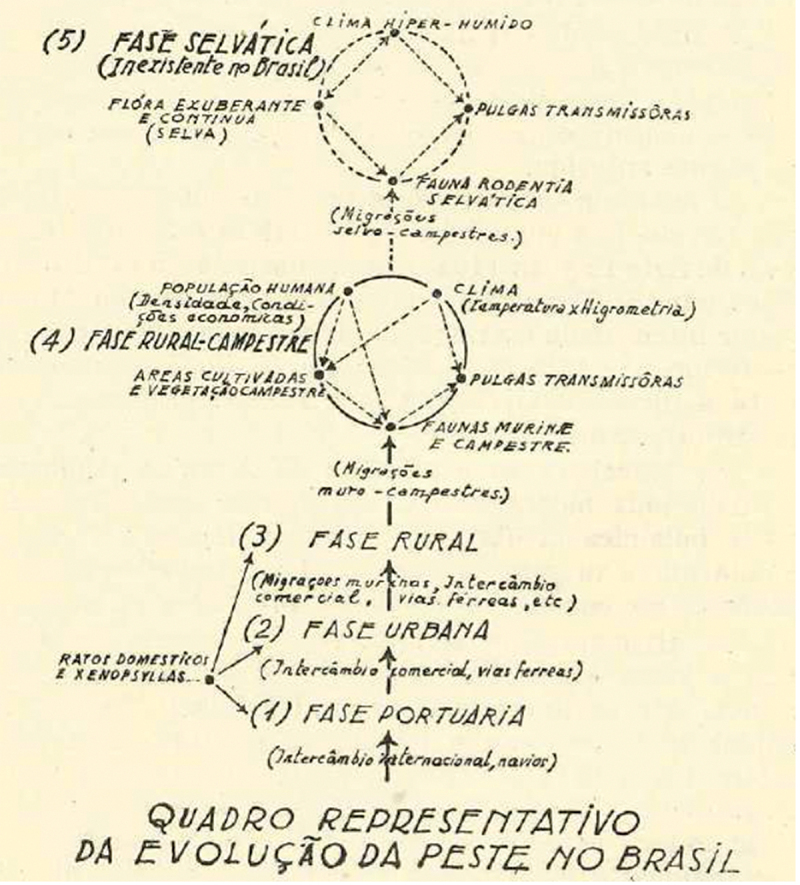
The evolution of plague in Brazil.

In 1951, Roland Simon, SNP doctor and chief of the SNP’s laboratory in Maceió, discussed in an official memoir the history of plague in Brazil and its possible evolution. Taking into consideration the work done by the SNP in the last decade and drawing upon Macchiavello’s reasoning on sylvatic plague, Simon (1951:23–25) proposed a scheme of the evolution of plague in Brazil based on five phases (see Figure 2): (1) port phase, (2) urban phase, (3) rural phase, (4) rural-country phase, and (5) sylvatic phase. According to Simon, in the first three phases, domestic rats and their parasites were *exclusively* responsible for spreading the plague to humans (Simon 1951:25). The fourth phase seemed to be emergent in the late 1940s, being characterized by intense cross-infections between rats and rodents at the vicinities of the rural area (Simon 1951:23–25). The last phase, the sylvatic one, remained absent, and, therefore, its characteristics could only be imagined. To Simon (1951:25) domestic rats would play no role in spreading this type of plague and the bacillus would circulate exclusively among “a murine sylvatic fauna” [*fauna rodentia selvática]*, living in a “hyper-humid climate” and in a landscape with “continuous and lush vegetation, the jungle [*selva*].” In this phase, human population were not affected by plague, and sylvatic plague was believed to exist completely independent from humanity (Simon 1951:25). Given these characteristics, Simon (1951:26) concluded that the Amazon “is the zone where it is more probable the appearance of the sylvatic plague” in Brazil.^6^

Simon’s diagram followed the general scheme devised by Jorge to explain the emergence of sylvatic plague reservoirs: from the coast to the wild, from domestic rats to wild rodents, from “culture” to “nature.” Simon adapted this schema to Brazil and categorized infection among wild rodents as sylvatic plague, conserved independently from domestic rats and humans, living in a *particular* landscape, the Amazon jungle. Therefore, the idea of sylvatic plague in Simon’s reasoning completely lost its attachment to the arid and semi-arid landscapes, which partly informed Jorge’s thinking in the 1920s, and became attached to the jungle. Three reasons could explain this shift. Firstly, the appearance of the idea of jungle yellow fever or sylvatic yellow fever in South America in the 1930s, which pointed to the existence of wild yellow fever reservoirs in forested zones (Emilio et al. 2008; Magalhães 2016:96–100). Secondly, the etymological connection between the word jungle and sylvatic in Portuguese. Thirdly, the long-standing representation of the Amazon as a vast uninhabited place full of wild animals, despite the human populations present there, indigenous and settlers alike. Thus, it made sense for a doctor from the urbanized Atlantic coast, like Simon, to imagine that sylvatic plague should be related to the Amazon jungle rather than any other Brazilian environment. Therefore, in Simon’s diagram, the locus of sylvatic plague appeared not only as something ontologically apart from humanity, but as geographically apart from the rest of Brazil. In sum, by a complex process of circulation, translation, and adaptation, in Brazil sylvatic plague became in the 1940s truly sylvatic, though rather as a prophecy, in the sense of an exclusive attachment to the *selva*.

## Conclusion

This article is the first in-depth study of the emergence, first usages and adaptations of the concept of sylvatic plague in the second quarter of the twentieth century. By examining it as a complex and global process that connected Portuguese and Brazilian imaginaries about the Amazon, research on rats and wild rodents in the first decades of the Third Plague Pandemic, the work of an international health agency, European imperialism in Africa, and sanitary campaigns in Brazil, the article has contributed to a critical understanding of the epistemological, political, and imperial histories of this concept. The article has provided a larger discussion of the idea of sylvatic diseases, contributing then to problematize our understanding of human-animal relationships and zoonosis. Contrary to the other sylvatic disease that was conceptualized a few years later – sylvatic yellow fever – sylvatic plague was characterized by an ambiguous connection with forests and jungles. While, on the one hand, the word sylvatic connoted this kind of environment, on the other hand, no sylvatic plague reservoir existed there. Therefore, the biggest and most controversial question around the concept of sylvatic plague was the definition of the space occupied by its reservoir. As has been shown, this space changed thorough time. In some iterations, this space seemed to be restricted to a particular kind of landscape – a desert or a jungle, for instance – while in others it pointed to a general uninhabited space. But some agreements seemed to exist, namely the idea that sylvatic plague was perpetuated among wild rodents in wild places. Henceforth, when the concept of sylvatic plague was used, it produced a separation between culture and nature and framed the second as the locus where the sylvatic plague reservoir existed independently from humans and domestic rats. But in so doing, the concept of sylvatic plague suggested nonetheless a larger idea of society, composed not only of humans, but of humans *and* domestic rats. The concept of sylvatic plague thus ended up reifying but also to unsettling the nature-culture dichotomy.

## Notes

1. Reasons for the expansion of the OIHP in the 1920s spanned from the necessity of including countries already represented in the LNHO (Office International d’Hygiène Publique 1924:7) to disputes between France and Great Britain, since the arrival of the colonies represented more votes to positions sustained by the metropoles (Office International d’Hygiène Publique 1933:5–6; Permanent Committee of the Office International d’Hygiène Publique 1925:fol. 121).

2. In a different vein, the Japanese delegate Mitsuzo Tsurumi argued that the role of the tarbagan on conserving plague bacilli in Manchuria was not proved. Jorge acknowledged this criticism and the hypothesis that human migrations could be behind plague outbreaks in Manchuria. Nevertheless, Jorge maintained his position (Office International d’Hygiène Publique 1926:119–120).

3. The book also included all the local reports, and a synoptical study on flea fauna and plague written by Émile Roubaud (Jorge 1928).

4. The conference was organized by South Africa and sponsored by the LNHO. It assembled mainly British and Portuguese colonies in Africa, and British India as an observer. Initially planned to deal with concerns that aerial traffic could spread mosquitos and yellow fever, the conference also focused on problems such as plague among wild rodents, but the term “sylvatic plague” was not used then. On this conference see (Borowy 2009:225–230).

5. Jorge’s ideas appeared for the first time in English in Clare Stallybrass’ handbook *Principles of Epidemiology*. In this book, sylvatic plague was defined as “the reservoir among wild rodents, spreading slowly from colony to colony, independent of man’s lines of communication, mainly affecting country of a steppe or veld-like character. To this type of epizootic, Jorge has given the title of selvatic plague [sic]” (Stallybrass 1931:310).

6. Simon (1951:26) writes the “Amazon district” following a zoogeographical division of South America in 11 districts developed by the Argentinian zoologists Angel Cabrera and José Yepes. In Cabrera and Yepes (1940:14), the Amazon district corresponded to “the central part of Brazil with the basins of the Amazon and the Madeira [Rivers], from the Guianas and the Atlantic Coast until the eastern part of Bolivia, Peru, Ecuador, and Colombia.”
